# Insulin in Myenteric Neurons: Time-Dependent and Regional Changes in Type 1 Diabetic Rats

**DOI:** 10.3390/cells14110809

**Published:** 2025-05-30

**Authors:** Abigél Egyed-Kolumbán, Benita Onhausz, Bence Pál Barta, Zita Szalai, Ildikó Huliák, Mónika Kiricsi, Mária Bagyánszki, Nikolett Bódi

**Affiliations:** 1 Department of Physiology, Anatomy and Neuroscience, Faculty of Science and Informatics, University of Szeged, Közép Fasor 52, H-6726 Szeged, Hungary; egyed.abigel@bio.u-szeged.hu (A.E.-K.); onhausz.benita@bio.u-szeged.hu (B.O.); barta.bence@bio.u-szeged.hu (B.P.B.); zszalai@bio.u-szeged.hu (Z.S.); bmarcsi@bio.u-szeged.hu (M.B.); 2 Department of Biochemistry and Molecular Biology, Faculty of Science and Informatics, University of Szeged, Közép Fasor 52, H-6726 Szeged, Hungary; huliakildiko@bio.u-szeged.hu (I.H.); kiricsim@bio.u-szeged.hu (M.K.)

**Keywords:** animal models, hyperglycemia, insulin, myenteric neurons, neuronal insulin uptake, neuronal nitric oxide synthase, type 1 diabetes

## Abstract

Enteric neurons regulating motility display regional damage to diabetes. By inhibiting neuroinflammation, insulin can contribute to neuronal survival, therefore, we aimed to investigate the presence of insulin in myenteric neurons and their nitrergic population in acute and chronic rat models of type 1 diabetes. One or ten weeks after the onset of hyperglycemia, gut segments and the pancreas of control, diabetic, and insulin-treated diabetic rats were investigated. In the controls, insulin-immunoreactive neurons comprised 8–9% of the total myenteric neuronal population in the ileum and colon and 2–4% in the duodenum. Except for the duodenum, this proportion was significantly increased in acute hyperglycemic rats and was decreased in the colon of the chronic ones. However, the proportion of insulin-immunoreactive nitrergic neurons remained unchanged in all segments in chronic hyperglycemia. Immunogold electron microscopy revealed an increased density of insulin-labelling gold particles in diabetic duodenal ganglia of the chronic experiment. Insulin mRNA was not detected in intestinal samples either in controls or diabetics. These findings support time-dependent and regional alterations in the proportion of insulin-immunoreactive myenteric neurons and their nitrergic subpopulation. Regionally different insulin content of myenteric neurons may contribute to their protection from diabetic damage.

## 1. Introduction

Insulin impact on glucose metabolism is crucial to maintain cellular homeostasis. Insulin deficiency results in high blood glucose concentration and energy deficit in cells originating from inappropriate glucose uptake [1]. Long-lasting hyperglycemia accompanying type 1 diabetes leads to a range of pathological processes including increased oxidative stress and impaired antioxidant defense [2].

Using an experimentally induced type 1 diabetic animal model, we have investigated for many years the diabetic damages of enteric neurons and intestinal milieu and the effects of exogenous insulin replacement in their prevention [3]. Although not all diabetic alterations can be prevented by insulin treatment, its beneficial role is not negligible. The diabetic induction of intestinal inflammatory pathway elements, among others, was prevented by insulin treatment [4]. The anti-inflammatory effects of insulin have also been shown in other diseases in both animal experiments and human studies [5,6]. By inhibiting neuroinflammation and cell death, insulin exerts a neuroprotective action and has a role in synaptic plasticity [7,8].

It is well-known that the pathogenesis of type 1 diabetes leads to the serious destruction of pancreatic islets, which are the main sources of insulin production. However, extrapancreatic insulin expression has been reported in several organs and different species. Liver, bone marrow, or adipose tissue are among the organs which are able to synthesize insulin in diabetic models [9,10]. Insulin transcripts and protein were also found in different brain regions, like the cortex, hippocampus or hypothalamus [11]. Moreover, insulin acts on brain neurons expressing neurotransmitters such as serotonin, gamma-amino-butyric acid, or acetylcholine which highlights the impact of insulin in central neurotransmission regulation [12,13,14]. Based on these observations, the question of whether there is insulin expression in enteric neurons has been raised. The close functional link between the pancreas and the proximal small intestine as well as their physical proximity further emphasize the importance of this issue. That pancreas-projecting enteric neurons via their cholinergic and serotonergic projections may have an impact on pancreatic hormone production [15,16] has been an understudied topic. Enteroendocrine cells located in intestinal epithelium and pancreatic beta-cells show similarities in their gene expression profiles during embryonic differentiation [17]. This was further supported by Egozi et al. [18], identifying high level of insulin expression in enteroendocrine K/L cells in human fetuses during development.

Gut regional sensitivity of myenteric neurons to hyperglycemic insults is evidenced, moreover, as distinct subpopulations are vulnerable in different ways to diabetic damage [4,19]. Among them, nitrergic neurons represent a very significant subpopulation. Their proportion within myenteric neurons can reach 50%. These neurons take part in the regulation of the descending inhibition of peristalsis and are highly susceptible to diabetic damage [20], which results in severe motility disturbances [19]. The underlying mechanisms are really complex and involve a varied induction of inflammatory and antioxidant pathways in myenteric neurons located in different gut segments [3], and this issue needs further investigation. It should be noted that insulin may exert distinct effects on neuronal subpopulations with different neurochemical phenotypes therefore insulin effects are closely related to its receptor signaling [21]. Insulin receptors are expressed on both central and peripheral neurons [21,22], furthermore, enteric neurons are also sensitive to insulin [23]. Considering the effects of insulin on neuronal survival, our primary aim was to elucidate whether there is endogenous insulin expression in myenteric neurons and their nitrergic neuronal subpopulation, which is particularly affected in diabetic motility dysfunction. If the presence of insulin in enteric neurons is verified, we also plan to investigate its hyperglycemic alterations from segment to segment in both acute and chronic type 1 diabetic rat models.

## 2. Materials and Methods

### 2.1. Acute and Chronic Type 1 Diabetic Animal Models

Adult male Wistar rats (Toxi-Coop Zrt., Balatonfüred, Hungary) given standard laboratory chow (Innovo Kft., Zsámbék, Hungary) and drinking water ad libitum, were used in our study.

**Figure 1:**
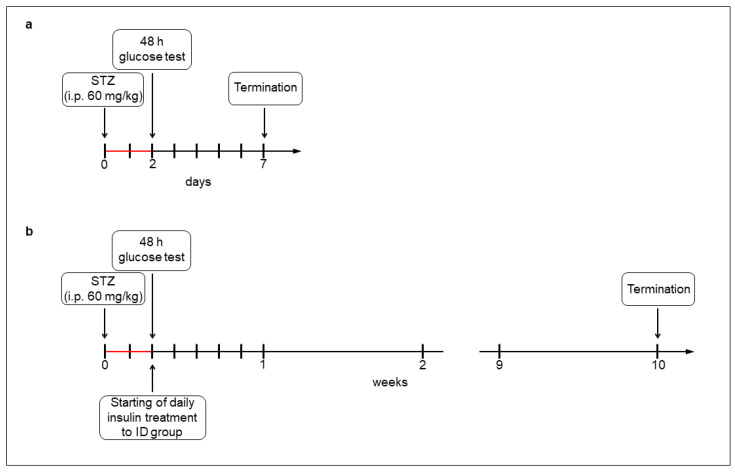
Experimental setup of acute (**a**) and chronic (**b**) models of type 1 diabetic rats. Hyperglycemia was induced by a single streptozotocin injection (i.p. 60 mg/kg). After 48 h, the non-fasting blood glucose concentration was determined and the animals were considered diabetic if it was higher than 18 mmol/L. In the chronic experiment, one group of hyperglycemic rats received insulin treatment twice a day.

For the acute (1-week) hyperglycemic experiments, the rats (200–250 g) were divided randomly into streptozotocin (STZ)-induced 5-day diabetic (acute diabetics, n = 8), and age-matched control (acute controls, n = 6) groups. Hyperglycemia was induced by a single injection of STZ (i.p. 60 mg/kg, Sigma-Aldrich, Budapest, Hungary) [4,19], while the control group got vehicle. After 48 h, the non-fasting blood glucose concentration was determined, and the animals were considered diabetic if it was higher than 18 mmol/L [4,19]. Weight and glycemic parameters of rats were monitored daily under the 1-week experiment (Figure 1a).

For the chronic (10-week) hyperglycemic experiments, the rats (180–250 g) were divided randomly into STZ-induced 10-week diabetic (chronic diabetics; n = 16), insulin-treated STZ-induced diabetic (insulin-treated diabetics; n = 14), and sex- and age-matched control (chronic controls; n = 17) groups. Induction and criteria of hyperglycemia were the same as for the above mentioned. The insulin-treated group received injections of insulin (Humulin M3, Eli Lilly Nederland, Utrecht, The Netherlands) twice a day (3-3 IU, s.c.). Chronic diabetic and control rats received the same volumes of saline (s.c.). The blood glucose concentrations and animal weights were measured weekly (Figure 1b). Spontaneously recovered diabetic animals, or whose glucose level decreased under 18 mmol/L, were excluded from the study [4].

### 2.2. Tissue Handling

One or ten weeks after the onset of hyperglycemia, the animals were killed by cervical dislocation under chloral hydrate anesthesia (375 mg/kg i.p.). Different gut segments and the pancreas of all experimental groups of rats were dissected and rinsed in 0.05 M phosphate buffer (PB; pH 7.4), and were processed for fluorescent immunohistochemistry, quantitative immunogold electron microscopy, ELISA, and PCR. Intestinal samples were taken from the duodenum, ileum, and colon [4].

For fluorescent microscopy, the gut segments were cut along the mesentery, and fixed overnight in 4% formaldehyde solution buffered with 0.1 M PB (pH 7.4; 4 °C). After washing, the mucosa, submucosa, and circular smooth muscle were removed, and whole-mount preparations with the myenteric plexus were prepared. After fixation (4% formaldehyde solution, overnight at 4 °C), the pancreatic samples were embedded in paraffin for sectioning (5 µm). For post-embedding electron microscopy, small pieces (2–3 mm) of different gut segments and pancreas were fixed in 2% paraformaldehyde and 2% glutaraldehyde solution and then further fixed for 1 h in 2% OsO_4_. After rinsing in buffer and dehydrating in increasing ethanol concentrations and acetone, they were embedded in Embed812 (Electron Microscopy Sciences, Hatfield, PA, USA). For the ELISA and PCR, the 3 cm long gut segments were cut along the mesentery. After removing both the mucosa and submucosa, the intestinal smooth muscle containing the myenteric plexus and pancreatic samples were snap-frozen in liquid nitrogen and stored at −80 °C until use [4].

### 2.3. Fluorescent Immunohistochemistry

For double and triple-labelling immunohistochemistry [4], whole-mount preparations from different intestinal segments as well as pancreas paraffin sections were immunostained with different insulin, neuronal nitric oxide synthase (nNOS), and peripherin pan-neuronal markers. Briefly, after blocking in TBS containing 1% bovine serum albumin and 10% normal goat serum, the myenteric whole-mounts and pancreatic sections were incubated overnight with a combination of primary antibodies (Table 1) at 4 °C. After washing in TBS with 0.025% Triton X-100, whole-mounts and sections were incubated with secondary antibodies (Table 1) for 1 h at room temperature. No immunoreactivity was observed on negative control samples. Whole-mounts were mounted on slides in Fluoromount^TM^ aqueous mounting medium (Sigma-Aldrich, Budapest, Hungary), while sections were mounted in Fluoroshield^TM^ with DAPI mounting medium (Sigma-Aldrich, Budapest, Hungary), and observed and photographed with a fluorescent microscope (Zeiss Imager Z.2, Axiocam 506 mono camera, Zeiss, Jena, Germany) [4]. Fifty-eighty myenteric ganglia were taken from each intestinal segment from each experimental group of acute and chronic diabetic studies and the proportion of myenteric neurons that are immunoreactive (IR) for either insulin or insulin-nNOS were counted per ganglia. All neurons in the observed ganglia were evaluated. Insulin-immunostained pancreas sections were used as standards of insulin immunoreactivity.

### 2.4. Post-Embedding Immunogold Electron Microscopy

Three Embed blocks originating from different gut segments and pancreas of each group of chronic experiment were used to prepare 70 nm ultrathin sections, which were mounted on nickel grids for immunogold labelling [4]. Ultrathin sections (three grids per block) were incubated overnight in anti-insulin (Cat. No. I2018, Table 1) primary antibody, followed by colloidal gold conjugated anti-mouse IgG (conjugated to 18 nm gold particles; 115-215-071, Jackson ImmunoResearch, West Grove, PA, USA; final dilution 1:20) secondary antibody for 3 h. The specificity of the immunoreaction was assessed in all cases by omitting the primary antibody. Sections were counterstained with uranyl acetate (Merck Millipore, Darmstadt, Germany) and lead citrate (Merck Millipore, Darmstadt, Germany) and examined and photographed [4]. The quantitative features and the subcellular distributions of the insulin-labelling gold particles were determined in the myenteric ganglia and both endocrine and exocrine parts of the pancreas. Digital photographs numbering 50–80 of 7–10 myenteric ganglia per gut segment per condition, as well as 60–80 digital photographs of 5–6 Langerhans islets and 60–80 digital photographs of exocrine pancreas per condition were made at a magnification of 20,000× with a JEOL JEM 1400 transmission electron microscope (JEOL, Tokyo, Japan) and the TEM Center 1.6.9. software (JEOL, Tokyo, Japan). During quantification, the regions of interest including the area of myenteric ganglia/islet cells/exocrine pancreatic cells without nuclei were circled and the number of insulin-labelling gold particles was counted. The intensity of the labelling was expressed as the total number of gold particles per µm^2^.

### 2.5. Measurement of Tissue Insulin Concentration of Muscle/Myenteric Plexus Homogenates

Tissue samples of the chronic experiment, containing the intestinal smooth muscle with the myenteric plexus in between, were frozen in liquid nitrogen, crushed into powder, homogenized in 500 µL homogenizing buffer (100 µL protease inhibitor cocktail (Sigma-Aldrich, Budapest, Hungary) in 20 mL 0.05 M PB), and centrifuged (5000 rpm, 20 min, 4 °C) [4]. Quantitative ELISA was used to determine the insulin levels of the muscle/myenteric plexus homogenates according to the manufacturer’s instructions (GA-E0715RT; GenAsia Biotech Co., Ltd., Shanghai, China). Each sample was measured in duplicate to increase the reliability of the measurements. Optical density was measured at 450 nm (Benchmark Microplate Reader; Bio-Rad, Budapest, Hungary). Tissue insulin concentrations were expressed as mIU/mg protein.

Tissue protein content of intestinal samples was determined by a commercial protein assay kit. Bradford reagent was added to each sample (10 min incubation) and the samples were assayed spectrophotometrically at 595 nm. Protein level was expressed as mg protein/mL [4].

### 2.6. RNA Extraction, Reverse Transcription and PCR

Frozen intestinal (duodenum, ileum, colon) and pancreatic tissue samples of control and diabetic animals were pulverized under liquid nitrogen, homogenized in TRIzol reagent (Thermo Fischer Scientific, Waltham, MA, USA), and total RNA was isolated according to the manufacturer’s recommendations. RNA concentration and purity was assessed by NanoDrop ND-1000 spectrophotometer (Thermo Fischer Scientific, Waltham, MA, USA). Total RNA of 2 μg was reverse transcribed using TaqMan reverse transcription reagents (Thermo Fischer Scientific, Waltham, MA, USA) with random hexamer primers. To examine the potential expression of insulin, PCR reactions were carried out on pooled cDNA samples in 25 μL reaction volume with a temperature program of 95 °C for 5 min (initial denaturing), then 35 cycles of 10 s at 95 °C, 20 s at 62 °C, and 30 s at 72 °C. The RT-PCR reactions for each sample were performed in triplicate to increase the reliability of the measurements. PCR products were resolved on 2% agarose gel and visualized using SYBR Safe DNA Gel Stain (Thermo Fischer Scientific, Waltham, MA, USA). Glyceraldehyde 3-phosphate dehydrogenase (GAPDH) PCR reactions were used as internal controls. Insulin primers were designed by Primer3 software, and obtained from Merck Millipore (Darmstadt, Germany). GAPDH primers were a kind gift from Renáta Gáspár (University of Szeged, Szeged, Hungary). In order to optimize PCR conditions, we determined the proper primer concentration and the optimal annealing temperature. Primers were tested in the final concentration of 100–400 nM, while the annealing temperature for insulin amplification was optimized in gradient PCR in the range of 56–64 °C. Sequences of primers specific for insulin and GAPDH are shown in Table 2.

### 2.7. Statistical Analysis

The Kruskal–Wallis test with a Dunn’s multiple comparisons test were applied for data analysis by GraphPad Prism 6.0 (GraphPad Software, San Diego, CA, USA). The probability of *p* < 0.05 was set as the level of significance. All data were expressed as mean ± SEM.

## 3. Results

### 3.1. Weight and Glycemic Characteristics of Animals

The weight and blood glucose concentration of the rats of acute and chronic type 1 diabetic models are shown in Table 3. All rats of the acute and chronic experiments gained weight during the experimental period; however, its extent was smaller under chronic hyperglycemia. The average blood glucose level of hyperglycemic animals was almost five times higher than control values (29.1 ± 1.01 vs. 6.39 ± 0.12 mmol/L in acute, 27.88 ± 1.2 vs. 5.61 ± 0.11 mmol/L in chronic). Insulin treatment applied during the chronic experiment prevented the extremely high glucose concentrations (13.66 ± 0.91 mmol/L), but these were still higher than in the controls.

### 3.2. Proportion of Insulin-Immunoreactive Myenteric Neurons

**Figure 2:**
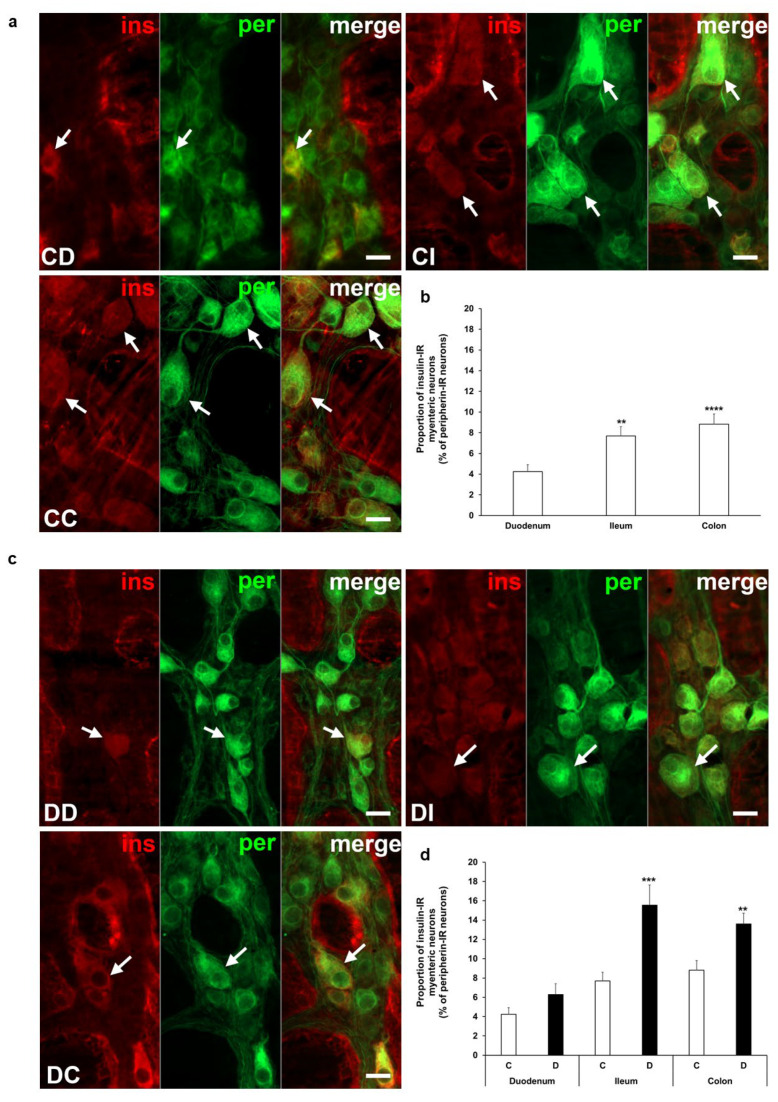
Representative fluorescent micrographs of whole-mount preparations of myenteric ganglia from the duodenum, ileum, and colon of control (**a**) and acute diabetic rats (**c**) after insulin-peripherin double-labelling immunohistochemistry. Peripherin as a pan-neuronal marker was applied to label myenteric neurons. Arrows—insulin-immunoreactive (IR) myenteric neurons, ins—insulin, per—peripherin. Scale bars: 20 μm. (**b**) Proportion of insulin-IR myenteric neurons in the duodenum, ileum, and colon of control rats of the acute experiment. The proportion of insulin-IR myenteric neurons was significantly higher in the distal parts compared to the duodenum. ** *p* < 0.01, **** *p* < 0.0001 (relative to control duodenum). (**d**) Proportion of insulin-IR myenteric neurons in the different gut segments of control and hyperglycemic rats of the acute experiment. In the acute hyperglycemics, the proportion of insulin-IR myenteric neurons was significantly increased in the ileum and colon. ** *p* < 0.01, *** *p* < 0.001 (relative to controls). Data are expressed as mean ± SEM. C—controls (n = 6 animals), D—acute diabetics (n = 8 animals). CD—control duodenum, CI—control ileum, CC—control colon, DD—diabetic duodenum, DI—diabetic ileum, DC—diabetic colon.

Insulin immunoreactivity of neurons within the ganglia of the myenteric plexus was revealed by fluorescent immunohistochemistry along the intestinal tract in both the acute and chronic diabetic animal models. Quantification of these myenteric neurons displays segmental differences in the investigated gut regions. Insulin-IR neurons showed the lowest proportion in the duodenum with 4% and it was about double in the ileum (7.7 ± 0.89%, *p* < 0.01) and colon (8.83 ± 0.98%, *p* < 0.0001) of control rats from the acute experiment (Figure 2a,b). In the acute hyperglycemic rats, the ratio of insulin-IR myenteric neurons markedly increased in the ileum and colon compared to controls (15.56 ± 2.08% vs. 7.7 ± 0.89%, *p* < 0.001 and 13.6 ± 1.12% vs. 8.83 ± 0.98%, *p* < 0.01, respectively), but remained unchanged in the duodenum (Figure 2c,d).

**Figure 3:**
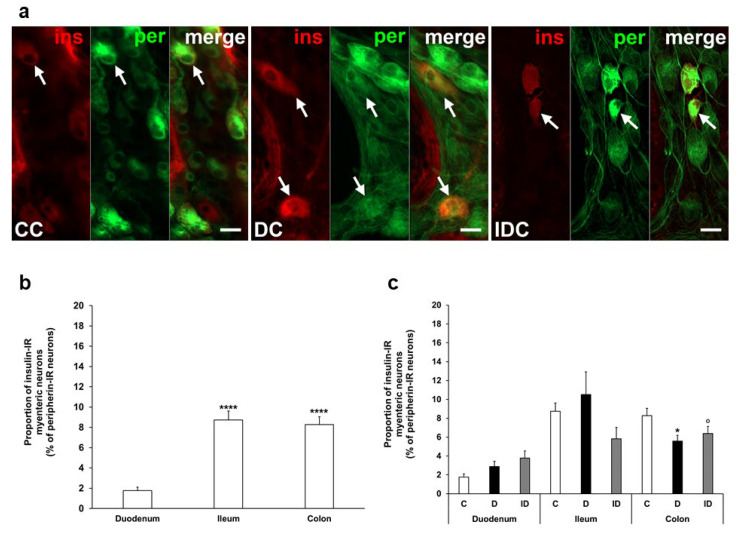
(**a**) Representative fluorescent micrographs of whole-mount preparations of myenteric ganglia from the colon of control, chronic diabetic, and insulin-treated diabetic rats after insulin-peripherin double-labelling immunohistochemistry. Peripherin as a pan-neuronal marker was applied to label myenteric neurons. Arrows—insulin-immunoreactive (IR) myenteric neurons, ins—insulin, per—peripherin. Scale bars: 20 μm. (**b**) Proportion of insulin-IR myenteric neurons in the duodenum, ileum, and colon of control rats of the chronic experiment. The proportion of insulin-IR myenteric neurons was significantly higher in the ileum and colon compared to the duodenum. **** *p* < 0.0001 (relative to control duodenum). (**c**) Proportion of insulin-IR myenteric neurons in the different gut segments of control, diabetic and insulin-treated diabetic rats of the chronic experiment. In the diabetics, the proportion of insulin-IR myenteric neurons was decreased in the colon. * *p* < 0.05 (relative to controls), ^o^ *p* < 0.05 (between diabetics and insulin-treated diabetics). Data are expressed as mean ± SEM. C—controls (n = 8 animals), D—diabetics (n = 9 animals), ID—insulin-treated diabetics (n = 7 animals). CC—control colon, DC—diabetic colon, IDC—insulin-treated diabetic colon.

In our chronic experiment, the insulin-IR neuronal proportion compared to the total number of myenteric neurons was also higher in the distal gut segments (8–9%) than in the duodenum (below 2%) of controls (Figure 3a,b). In diabetic rats, this proportion was significantly decreased in the colon (5.58 ± 0.61 vs. 8.27 ± 0.78, *p* < 0.05) without any changes in duodenal and ileal ganglia. Immediate insulin treatment failed to prevent the decrease in the percentage of insulin-IR myenteric neurons in the colon (Figure 3a,c).

**Figure 4:**
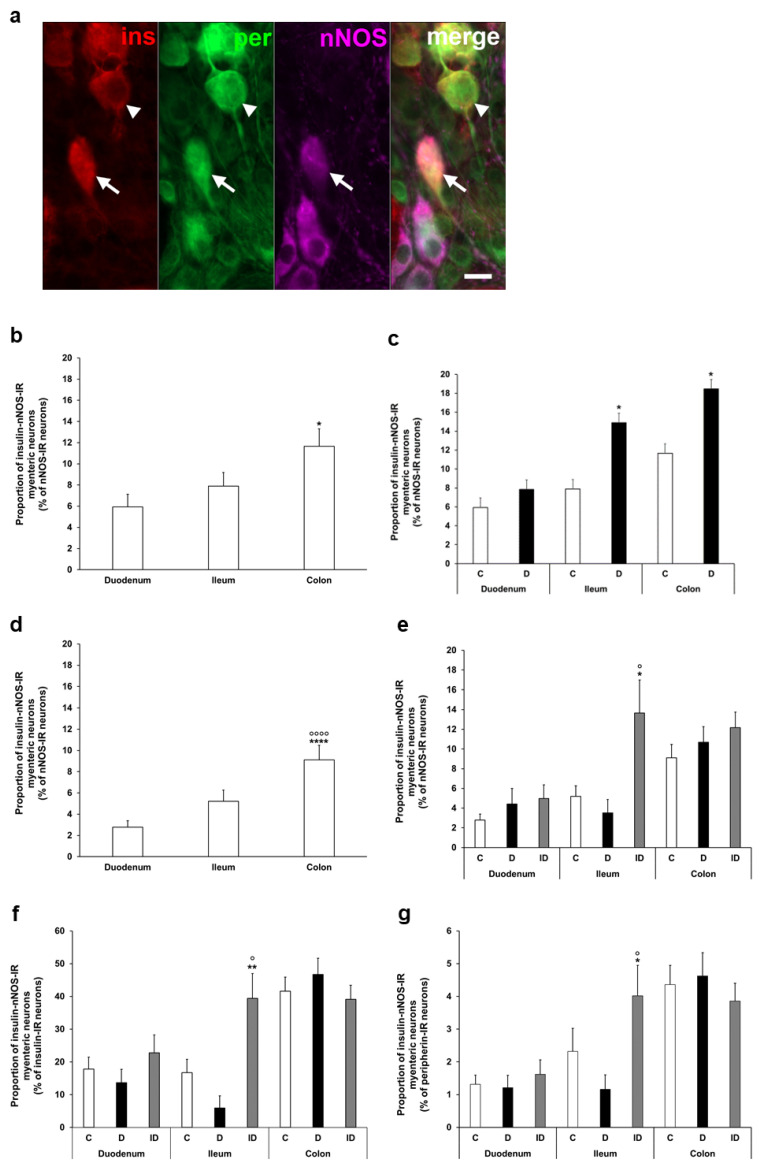
(**a**) Representative fluorescent micrograph of a whole-mount preparation of myenteric ganglia from the colon of insulin-treated diabetic rat after insulin-nNOS-peripherin triple-labelling immunohistochemistry. Peripherin as a pan-neuronal marker was applied to label myenteric neurons. Arrow—insulin-nNOS-immunoreactive (IR) myenteric neuron, arrowhead—insulin-IR myenteric neuron, ins—insulin, per—peripherin, nNOS—neuronal nitric oxide synthase. Scale bar: 20 μm. (**b**) Proportion of insulin-nNOS-IR myenteric neurons (per nNOS-IR neurons) in the duodenum, ileum, and colon of control rats of the acute experiment. This proportion was significantly higher in the colon compared to the small intestine. * *p* < 0.05 (relative to control duodenum). (**c**) Proportion of insulin-nNOS-IR myenteric neurons (per nNOS-IR neurons) in the different gut segments of control and hyperglycemic rats of the acute experiment. In the acute hyperglycemic rats, the proportion of insulin-nNOS-IR myenteric neurons was significantly increased in the ileum and colon. * *p* < 0.05 (relative to controls). (**d**) Proportion of insulin-nNOS-IR myenteric neurons (per nNOS-IR neurons) in the duodenum, ileum, and colon of control rats of the chronic experiment. This proportion was significantly higher in the colon compared to the small intestine. **** *p* < 0.0001 (relative to control duodenum), ^oooo^ *p* < 0.0001 (relative to control ileum). (**e**) Proportion of insulin-nNOS-IR myenteric neurons (per nNOS-IR neurons) in the different gut segments of control, diabetic and insulin-treated diabetic rats of the chronic experiment. This proportion did not change in diabetic rats, while insulin treatment increased the ratio of these neurons in the ileum. * *p* < 0.05 (relative to controls), ^o^ *p* < 0.05 (between diabetics and insulin-treated diabetics). (**f**) Proportion of insulin-nNOS-IR myenteric neurons (per insulin-IR neurons) in the different gut segments and conditions of the chronic experiment. This proportion was higher than 40% in the colon, and lower than 20% in the duodenum and ileum without significant changes in the diabetic rats. ** *p* < 0.01 (relative to controls), ^o^ *p* < 0.05 (between diabetics and insulin-treated diabetics). (**g**) Proportion of insulin-nNOS-IR myenteric neurons (per total myenteric neurons) in the different gut segments and conditions of the chronic experiment. This proportion was less than 5% in all of the investigated gut segments and it did not alter in diabetics. * *p* < 0.05 (relative to controls), ^o^ *p* < 0.05 (between diabetics and insulin-treated diabetics). Data are expressed as mean ± SEM. C—controls (acute: n = 6, chronic: n = 8 animals), D—diabetics (acute: n = 8, chronic: n = 9 animals), ID—insulin-treated diabetics (n = 7 animals).

To determine the ratio of insulin-IR nitrergic neurons within the myenteric ganglia, triple-labelling immunofluorescence was applied (Figure 4a). Quantification of enteric neurons containing both insulin and nNOS also demonstrated regional variations in the acute and chronic diabetic models. The proportion of insulin-nNOS-IR neurons per total number of nNOS neurons was highest in the colon of acute control animals (nearly 12%), and a significant increase was displayed in the ileum and colon of the acute hyperglycemic group (14.9 ± 2.73 vs. 7.89 ± 1.29%, *p* < 0.05; 18.46 ± 2.05% vs. 11.65 ± 1.66%, *p* < 0.05, respectively) (Figure 4b,c).

In control rats of the chronic experiment, the proportion of insulin-nNOS-IR neurons (per total nNOS neurons) also showed the highest value in colonic myenteric ganglia (9.12 ± 1.36%), being markedly higher than in small intestinal segments (duodenum: 2.78 ± 0.62%, ileum: 5.21 ± 1.06, *p* < 0.0001) (Figure 4d). However, no significant alterations were observed in this proportion in either gut segments of chronic diabetics (Figure 4e), while insulin treatment increased the ratio of these neurons in the ileum (Figure 4e).

From another point of view, among the total insulin-IR neurons, a higher value than 40% of insulin-nNOS-IR neurons was demonstrated in the colon, and this value was lower than 20% in the duodenum and ileum (Figure 4f). However, its percentage was less than 5% in all investigated gut segments (duodenum: 1.32 ± 0.28, ileum: 2.32 ± 0.71, colon: 4.36 ± 0.59) when compared to the total myenteric neuronal number, and it did not change significantly in chronic diabetics (Figure 4g).

### 3.3. Subcellular Localization and Distribution of Insulin Within the Ganglia

**Figure 5:**
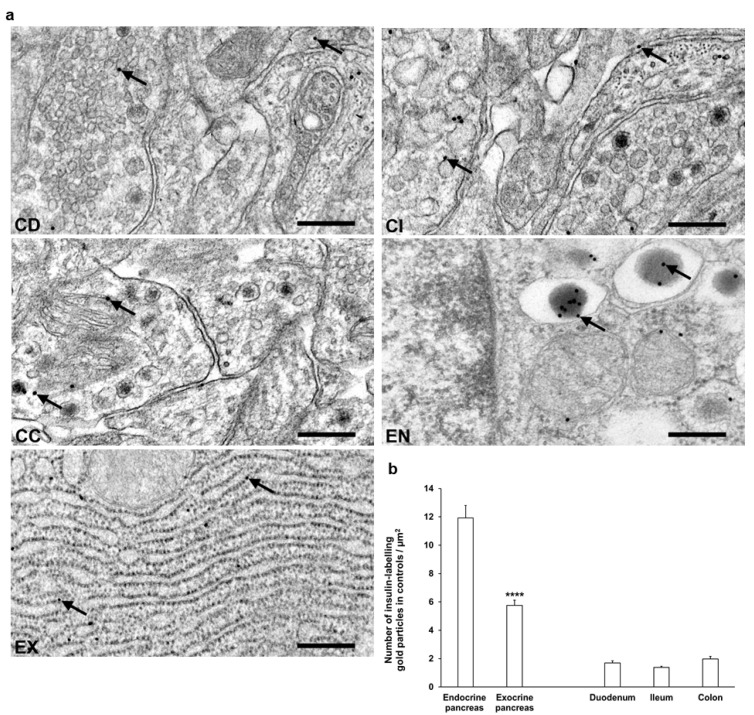
(**a**) Representative electron micrographs of portions of myenteric ganglia from duodenum (CD), ileum (CI), and colon (CC) as well as endocrine (EN) and exocrine (EX) pancreas of control rats after insulin post-embedding immunohistochemistry. Arrows—18 nm gold particles labelling insulin. Scale bars: 100 nm. (**b**) Quantitative evaluation of gold particles labelling insulin in myenteric ganglia from different gut segments and pancreas of control rats. All gut segments represented similarly low number of insulin-labelling gold particles relative to 6 times more gold labels in endocrine pancreas and also 3 times more gold labels in the exocrine part. **** *p* < 0.0001 (between endocrine and exocrine pancreas). Data are expressed as means ± SEM. n = 6 animals.

Transmission electron microscopy was used for the evaluation of subcellular insulin distribution in myenteric ganglia and in pancreas as internal standard in our chronic type 1 diabetic rat model (Figure 5a). The majority of insulin-labelling gold particles were localized to vesicles within cells or bound to plasma membrane in myenteric ganglia (Figure 5a).

In control animals, a low, similar density of insulin-labelling gold particles was observed in all gut segments. When comparing the density to the pancreatic sections, the insulin immunogold labels in the different intestinal segments were 6–8 times lower than endocrine pancreas and 3–4 times lower than exocrine pancreas (Figure 5b).

**Figure 6:**
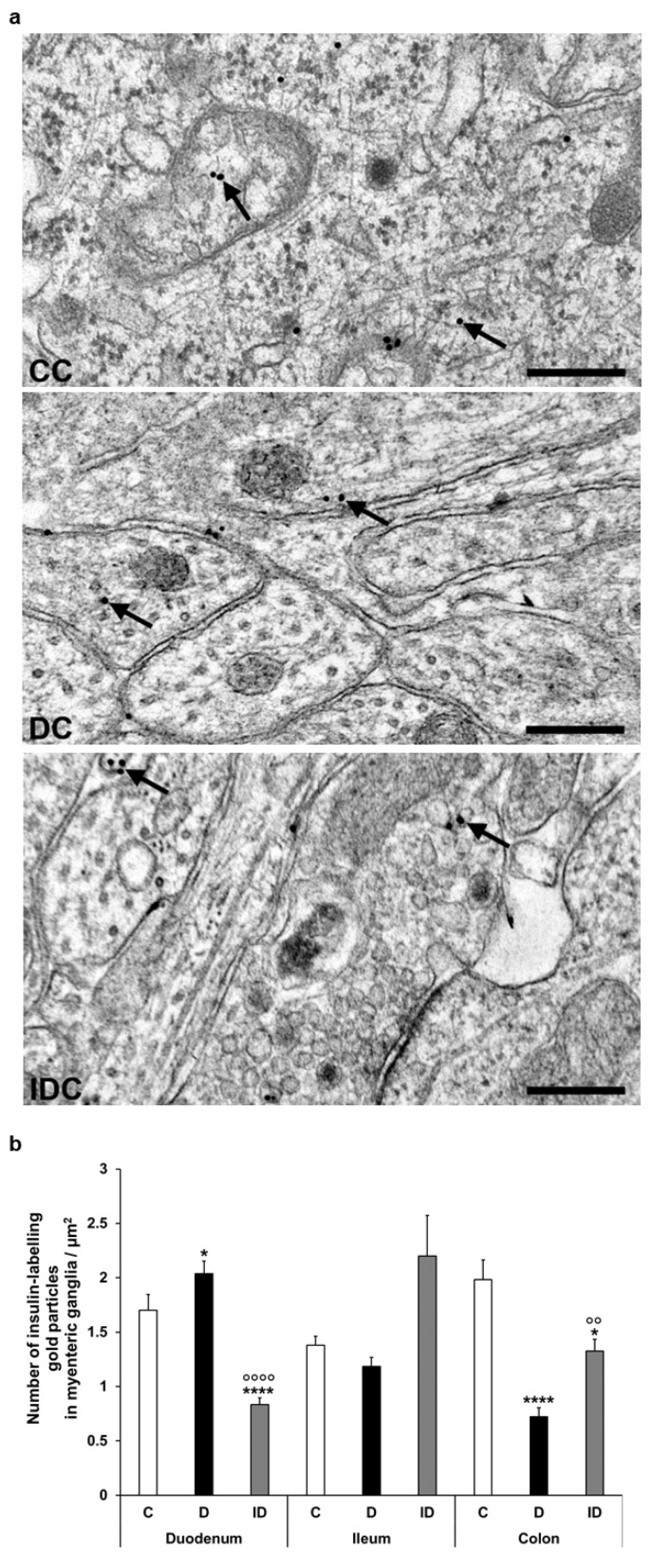
(**a**) Representative electron micrographs of different parts of myenteric ganglia from colon of control (CC), diabetic (DC), and insulin-treated diabetic (IDC) rats after insulin post-embedding immunohistochemistry. Arrows—18 nm gold particles labelling insulin. Scale bars: 100 nm. (**b**) Quantitative evaluation of gold particles labelling insulin in myenteric ganglia from different gut segments and conditions. The number of insulin-labelling gold particles increased in the duodenum and decreased in the colon. * *p* < 0.05, **** *p* < 0.0001 (relative to controls), ^oo^ *p* < 0.01, ^oooo^ *p* < 0.0001 (between diabetics and insulin-treated diabetics). Data are expressed as means ± SEM. C—controls (n = 6 animals), D—diabetics (n = 6 animals), ID—insulin-treated diabetics (n = 4 animals).

Despite the notable decrease in insulin density in both the endocrine and exocrine pancreas of diabetics (2.75 ± 0.18 vs. 11.93 ± 0.88 gold particles/µm^2^, *p* < 0.0001 and 4.39 ± 0.21 vs. 5.76 ± 0.36 gold particles/µm^2^, *p* < 0.01, respectively), the number of insulin-labelling gold particles showed different changes from intestinal segment to segment. It increased in the duodenum (*p* < 0.05), remained unchanged in the ileum, and robustly decreased in colonic ganglia (*p* < 0.001) of diabetic rats. Insulin treatment was partially effective in the colon; however, it resulted in a significant decrease of gold particle density to less than the control value in the duodenum (Figure 6).

### 3.4. Insulin Protein and mRNA Concentration in Intestinal Homogenates

**Figure 7:**
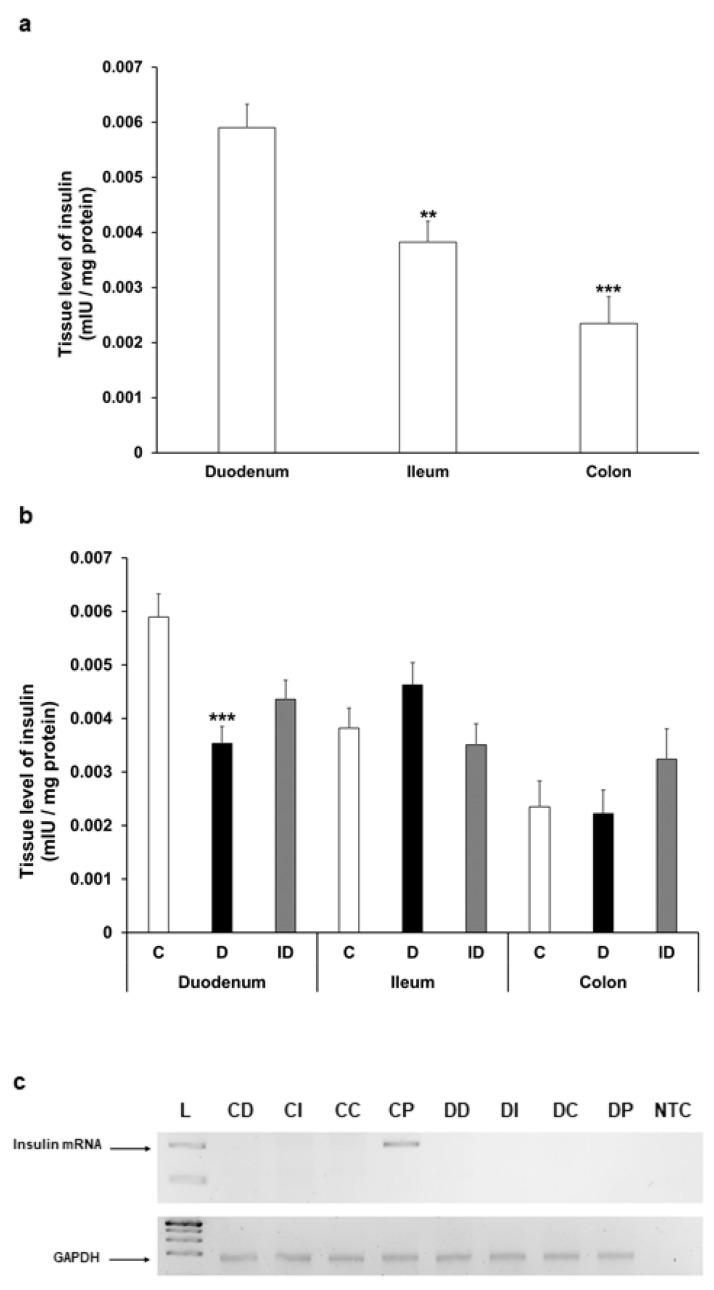
(**a**) Tissue levels of insulin in intestinal smooth muscle layer homogenates including the myenteric plexus from the duodenum, ileum, and colon of control rats. The insulin tissue level was highest in the duodenum. ** *p* < 0.01, *** *p* < 0.001 (relative to control duodenum). (**b**) Tissue levels of insulin in intestinal homogenates from different gut segments and conditions. The tissue insulin level was significantly decreased only in the diabetic duodenum. *** *p* < 0.001 (relative to controls). Data are expressed as means ± SEM. C—controls (n = 8 animals), D—diabetics (n = 6 animals), ID—insulin-treated diabetics (n = 8 animals). (**c**) Relative levels of insulin and GAPDH mRNA in tissue homogenates from different gut segments and pancreas of control (n = 3) and diabetic (n = 3) rats. Insulin mRNA was only detected in the pancreas of controls (size of PCR product is 206 bp). Endogenous control GAPDH expressed consistently in all rat tissue samples. L—DNA ladder, CD—control duodenum, CI—control ileum, CC—control colon, CP—control pancreas, DD—diabetic duodenum, DI—diabetic ileum, DC—diabetic colon, DP—diabetic pancreas, NTC—non-template control.

The insulin level showed a decreasing tendency along the intestine in gut smooth muscle/myenteric plexus tissue homogenates of control rats; it was highest in the duodenum, more than 30% lower in the ileum (*p* < 0.01), and 60% lower in the colon (*p* < 0.001) (Figure 7a). In diabetic rats, the tissue concentration of insulin altered only in the duodenum, where it significantly decreased compared to control (0.0059 ± 0.0004 mIU/µg vs. 0.0035 ± 0.0003 mIU/µg, *p* < 0.001) (Figure 7b).

To examine the possibility of intestinal insulin expression, reverse transcription followed by conventional PCR reactions were carried out on RNA samples isolated from the duodenum, ileum, colon, or pancreatic tissues of control and diabetic animals. PCR products were resolved on 2% agarose gel and visualized by DNA-binding fluorescent dye. Our aim was to uncover whether there was any mRNA corresponding to in situ insulin expression in these tissue samples, and in case of insulin expression could be conformed; then, the examination would proceed with a more detailed quantitative real-time PCR approach to compare the expression levels between the samples. As expected, in the pancreatic samples of control animals the expression of insulin could be verified; however, no insulin mRNA expression was observed in the duodenum, ileum, or colon samples of the control rats (Figure 7c). Not surprisingly, we did not detect insulin mRNA in the samples obtained from the pancreas of diabetic animals; thus, the absence of its expression further validated the diabetic condition. Importantly, no sign of in situ insulin production was noticed in diabetic animals along the intestinal tract (Figure 7c). Based on these results indicating the lack of in situ insulin production, there was no point in proceeding to a quantitative real-time PCR examination on these samples.

## 4. Discussion

To our knowledge, this is the first study to investigate insulin expression in the enteric nervous system. We showed that a subset of myenteric neurons contains insulin, and insulin presence displays imparity in different gut segments. Based on our immunofluorescence study, 8–9% of myenteric neurons was insulin-IR in distal segments, while this value was less than half in the proximal small intestine in healthy rats. We do not rule out the possibility that oxidative stress in the distal gut [3,24] may contribute to higher insulin content, which could play a protective role in this region. Insulin concentration in the brain also shows regional differences, e.g., higher concentration of IR insulin was measured in the hypothalamus and olfactory bulb compared to other brain regions [25].

Myenteric neuronal insulin immunoreactivity was markedly influenced by hyperglycemia in our diabetic models. However, the duration of the hyperglycemic insult affected the proportion of the insulin-containing neuronal population. In our acute model, this proportion was distinctly increased in the ileum and colon of hyperglycemic rats. Nevertheless, during long-lasting hyperglycemia, the insulin-IR neuronal proportion did not change in the ileum and significantly decreased in the colon of diabetic rats. Interestingly, the lowest number of insulin-IR neurons in the duodenum remained unchanged both in our acute and chronic models. These findings also strengthen the observation that distal gut regions are more vulnerable to diabetic damage than the proximal part [3]. We also observed the highest baseline insulin level in duodenal tissue homogenates, which may also contribute to a favorable intestinal milieu and a lower susceptibility of the duodenum to hyperglycemic injury. The homogenates used included the myenteric ganglia and mostly smooth muscle from the gut wall. Intestinal smooth muscle is well vascularized, therefore, considering the proximity of the pancreas, the insulin in the tissue may also be of vascular origin. Insulin binding leads to both gut smooth muscle relaxation and nitric oxide/cyclic GMP pathway-mediated glucose transport stimulation [26,27]. Diabetes-related decrease in tissue insulin content was observed only in the duodenum, which also reflects segment-dependent alterations of the muscular environment.

On the other hand, the time-dependent alterations at the distal gut may refer to a prompt defense mechanism in the acute phase of hyperglycemia in which the number of insulin-IR myenteric neurons rapidly increased in response to the cessation of pancreatic insulin production. In this study, insulin mRNA expression was not detected in the intestine supposing the lack of gut insulin production. Therefore, the increased number of insulin-containing neurons presumably originates from enhanced insulin uptake from the environment. Others found that insulin is locally synthesized in neurogliaform interneurons of cerebral cortex [13] or in diabetic retina [28]. Interestingly, insulin mRNA was detected in the liver of different diabetic mice and rat models, but not in their non-diabetic counterparts [9]. Snyder et al. (2024) also verified that sucrose-stimulated enteroendocrine cells produced insulin which served as an activation signal for enteric neurons altering their excitability [23]. It is important to note that pharmacological conversion of exocrine pancreatic cells, gastric stem cells, intestinal epithelial cells, or gut endocrine progenitors into insulin-producing cells may offer a promising perspective for diabetes therapy [29,30,31,32,33].

After insulin binding, the activated insulin receptor initiates metabolic signaling cascades and is internalized by endocytosis [34], which is important in insulin uptake and clearance [35,36]. Distinct mechanisms, e.g., clathrin- or caveolae-mediated receptor endocytosis are characteristic of insulin transport in different brain regions or microvessels [37,38]. In this study, we also revealed that the majority of insulin-labelling gold particles were located in vesicles within myenteric neurons. Insulin receptors are widely expressed on different cell types including vascular endothelial cells and intestinal epithelium or brain neurons, emphasizing the role of insulin in glucose metabolism and intracellular signaling in health and disease [39,40,41,42,43,44,45]. Based on the above findings, the density and diabetic involvement of the neuronal insulin receptor in enteric ganglia should also be investigated in the future.

In our chronic experiments, the electron microscopic quantification of insulin-labelling gold particles confirmed the results of immunofluorescence in distal gut regions. However, in the duodenum, the density of these gold particles significantly increased in myenteric ganglia despite the unchanged number of insulin-IR myenteric neurons. This phenomenon may be explained by the enhanced insulin uptake of these neurons or the possible involvement of enteric glial cells in the observed changes [46]. It is not surprising that the pancreatic density of insulin gold labels is robust compared to its presence in enteric ganglia. However, this analysis gives a complex view of differences in its magnitude comparing the intestinal segments with pancreatic islets and the exocrine pancreas, which is also regulated by islet hormones [47].

As insulin-containing myenteric neurons located in different gut regions responded differently to diabetic damage, the effects of insulin treatment were segment-dependent. In the present study, insulin treatment was partially protective or ineffective against diabetic changes, or even caused significant changes when compared to controls. Although these observations are interesting, it is difficult to draw conclusions from them at this point in the research. Possible changes of functional insulin receptor level at the plasma membrane may be able to contribute to segment-dependent efficiency of insulin treatment. However, they do highlight the contradictory effects of insulin treatment, even when it maintains optimal blood glucose concentration levels.

In this study, insulin immunoreactivity in the nitrergic subpopulation demonstrated a similar increase as shown in the total myenteric neurons of acute hyperglycemic rats, but the proportion of insulin-IR nitrergic neurons remained unchanged in all gut segments of the chronic model. Cellek et al. [48] revealed that diabetic damage of nitrergic neurons can be different in the early and later stages of diabetes. Since the loss of nitrergic myenteric neurons is a hallmark of advanced diabetes [19], we hypothesize that insulin may help protect nNOS neurons that contain insulin. Insulin signaling in nitrergic neurons may modulate nNOS-dependent nitric oxide synthesis, as observed in hypothalamic neurons as well [49]. In addition, further investigations of other myenteric subpopulations are also required.

The novelty of our study is the focus on insulin presence in the enteric nervous system. The present findings have provided a comprehensive view about insulin immunoreactivity of myenteric neurons in different gut regions under control and diabetic states. However, further investigations are necessary to clarify the current observations. There is a possibility that low abundance intestinal insulin mRNA transcripts may be below the detection threshold; for instance, they may require verification by single cell sequencing. The mechanism of insulin uptake, the effects of insulin on myenteric neurons, and the reasons for segmental differences in insulin immunoreactivity also need further functional experiments in the future.

## 5. Conclusions

In conclusion, our study has shown that insulin immunoreactivity in myenteric neurons or their nitrergic subpopulation is gut segment-specific and highly influenced by hyperglycemia in a time-dependent manner. The absence of insulin mRNA in intestinal tissue indicates that myenteric neurons likely uptake insulin from their environment. Further studies are needed to understand how insulin affects enteric neuronal signaling.

## Figures and Tables

**Table 1:** Primary and secondary antibodies used in the experiments.

Primary Antibody	Host	Final Dilution	Product Code	Company
anti-peripherin polyclonal	rabbit	1:400	ab1530	EMD Millipore Corporation, Burlington, MA, USA
anti-nNOS polyclonal	guinea pig	1:500	432 005	Synaptic Systems GmbH, Goettingen, Germany
anti-insulin monoclonal IgG1	mouse	1:100	I2018	Sigma-Aldrich, Budapest, Hungary
anti-insulin monoclonal IgG2a kappa	mouse	1:200	IMG-7B2	ImmunoGenes Ltd., Budakeszi, Hungary
anti-insulin polyclonal IgG	rabbit	1:200	15848-1-AP	Proteintech ChromoTek GmbH, Planegg-Martinsried, Germany
anti-insulin polyclonal	guinea pig	1:100	ab7842	Abcam, Cambridge, UK
**Secondary Antibody**	**Host**	**Final** **Dilution**	**Product Code**	**Company**
anti-rabbit Alexa Fluor^®^ 488	goat	1:200	A11008	Life Technologies Corporation, Molecular Probes, Carlsbad, CA, USA
anti-guinea pig Cy5^®^	goat	1:200	ab102372	Abcam, Cambridge, UK
anti-mouse Cy^TM^3	goat	1:200	115-165-003	Jackson ImmunoResearch Laboratories, West Grove, PA, USA

**Table 2:** Sequences of primers used in PCR reactions.

Target	Forward Primer (5′-3′)	Reverse Primer (5′-3′)
insulin	GCCCTAAGTGACCAGCTACA	TAGAAGAATCCACGCTCCCC
GAPDH	GAAGGGCTCATGACCACAGT	GGATGCAGGGATGATGTTCT

**Table 3:** Weight and glycemic characteristics of the experimental animals in the acute and chronic models of type 1 diabetes.

ACUTE EXPERIMENT	Body Weight (g)		Blood Glucose Concentration (mmol/L)	
	Initial	Final	Initial	Final (Average)
Controls (n = 6)	219 ± 7.18	275 ± 9.21 *	6.83 ± 0.26	6.39 ± 0.12
Diabetics (n = 8)	216.5 ± 4.98	256.3 ± 8.19 *	5.91 ± 0.19	29.1 ± 1.01 ****^,^ °
**CHRONIC EXPERIMENT**	**Body Weight (g)**		**Blood Glucose Concentration (mmol/L)**	
	**Initial**	**Final**	**Initial**	**Final (Average)**
Controls (n = 17)	203.9 ± 3.17	432.2 ± 10.3 ****	5.09 ± 0.31	5.61 ± 0.11
Diabetics (n = 16)	206.8 ± 4.27	318.5 ± 11.94 *	5.38 ± 0.4	27.88 ± 1.2 ****^,^ °°°°
Insulin-treated diabetics (n = 14)	206.5 ± 4.77	446.8 ± 13.83 ****	5.08 ± 0.40	13.66 ± 0.91 ***^,^ °

Data are expressed as mean ± SEM; * *p* < 0.05, *** *p* < 0.001, **** *p* < 0.0001 vs. initial; ^o^ *p* < 0.05, ^oooo^ *p* < 0.0001 vs. final controls.

## Data Availability

The raw data supporting the conclusions of this article will be made available by the authors on request.

## Abbreviations

GAPDH	glyceraldehyde 3-phosphate dehydrogenase
IR	immunoreactive
nNOS	neuronal nitric oxide synthase
PB	phosphate buffer
STZ	streptozotocin
